# *Mycobacterium chelonae* soft‐tissue infection in an immunocompetent child

**DOI:** 10.1111/jpc.16310

**Published:** 2022-12-29

**Authors:** Alexis G Bosman, Jason Beer, Keith Grimwood

**Affiliations:** ^1^ Department of Paediatrics School of Medicine and Dentistry, Griffith University Gold Coast Queensland Australia; ^2^ Department of Orthopaedics, Gold Coast Health Gold Coast Queensland Australia; ^3^ Department of Infectious Diseases Gold Coast Health Gold Coast Queensland Australia

**Key Points**

1. *Mycobacterium chelonae* causes severe infections in both immunocompromised and healthy adults, some initially starting with cutaneous and soft infections, complicating inoculation injuries including tattooing and cosmetic procedures.
2. *M. chelonae* may also cause local infections in an immunocompetent child following an inoculation injury, appearing 4–8 weeks later, where no bacterial pathogens are cultured, any foreign body has been removed, and there is no improvement with antibiotics.
3. Treatment requires a combination of antibiotics for 4–6 months, and further surgical exploration and drainage of the wound is often necessary.

## Case Report

A previously healthy, fully immunised and immunocompetent 8‐year‐old child sustained a puncture wound to the dorsum of the left foot from a *Bidens pilosa* (cobbler's peg) plant while walking through the family's chicken coop. Entry point was in the webspace of toes 2/3. Three‐weeks post‐injury, erythema and a fluctuant swelling with a purulent discharge developed proximal to toes 2/3. The family doctor prescribed oral cefalexin, but when after a week there was little improvement, an ultrasound demonstrated a foreign body, 14 mm × 0.5 mm in dimension, between the necks of the second and third metatarsal bones. A referral was then made to our hospital for further management.

Four‐weeks post‐injury, an incision and drainage was undertaken to remove the plant material, and intra‐operative samples were sent for culture. No post‐operative complications were observed, and the patient was discharged home and advised to continue taking cefalexin for the next 10 days. Upon review 3‐days post‐surgery, the wound appeared clean without signs of infection, discharge or surrounding cellulitis, and intra‐operative cultures had not grown any organisms. However, 10 days post‐surgery mild pain returned and upon examination, the proximal end of the wound was observed to be open and discharging a small amount of serosanguinous fluid with mild erythema at the incision edges. Sixteen‐days post‐surgery, the laboratory reported a rapidly growing non‐tuberculous mycobacterial (NTM) species from the intra‐operative samples, which was identified (99.9%) as *Mycobacterium chelonae* by the state Mycobacterial Reference Laboratory using matrix‐assisted laser desorption/ionisation time‐of‐flight mass spectrometry (MALDI‐ToF‐MS). Susceptibility testing based upon the Clinical Laboratory Standards Institute clinical breakpoints found the isolate to be susceptible to clarithromycin (minimum inhibitory concentration 0.12 μg/mL), amikacin (4 μg/mL) and tobramycin (1.0 μg/mL); to have intermediate susceptibility to imipenem (16 μg/mL), ciprofloxacin (2.0 μg/mL) and moxifloxacin (2.0 μg/mL); and it was resistant to cefoxitin (>128 μg/mL), trimethoprim‐sulfamethoxazole (>8 μg/mL), doxycycline (>16 μg/mL) and linezolid (>32 μg/mL).

**Fig. 1:**
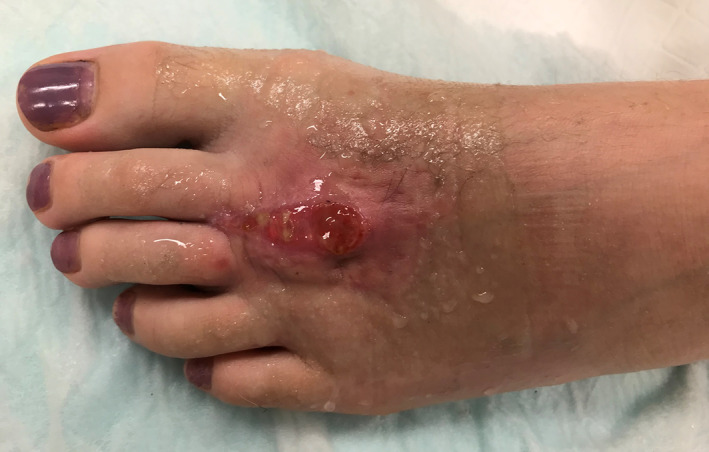
Infection site of *Mycobacterium chelonae* on dorsum of left foot.

When first seen at the Infectious Diseases Outpatient Clinic 3‐weeks following the operation and 7‐weeks after the initial injury, the child appeared well. There were no complaints of pain, fever or other constitutional symptoms. A small, non‐healing, moist granulomatous lesion at the proximal end of the incision site was observed (Fig. 1). The wound and surrounding skin were mildly erythematous, but there was no accompanying mass lesion or tenderness, and regional lymphadenopathy was not detected. The rest of the history and examination was unremarkable.

A decision was made to treat the infection medically. Once‐daily intravenous tobramycin 170 mg (6 mg/kg) was initiated and administered for 4‐weeks by our hospital‐in‐the‐home staff, additionally combined with single daily oral doses of azithromycin 250 mg (10 mg/kg) and moxifloxacin 400 mg (15 mg/kg). Apart from mild nausea and occasional diarrhoea, the treatment was well tolerated. However, after 4 weeks, there was little improvement in the appearance of the wound, which instead showed early signs of sinus formation with dermal breakdown, blistering and limited healing. Surgical wound debridement and scar tissue excision was then performed. A moderate amount of purulent material was found in the mid portion of the wound, which failed to culture bacteria, fungi or mycobacterial species. Because of the superficial nature of the infection, no imaging for bone involvement was done. Subsequently, the wound healed well, and a 6‐month course of oral azithromycin and moxifloxacin was completed without further incident. The child remains in good health >18 months after the original injury.

## Discussion

*Mycobacterium chelonae* is in the *M. chelonae–abscessus* complex of rapidly growing NTM species. It is found in soil, water and plants. Once thought to primarily infect immunocompromised patients resulting in severe, disseminated infections following invasive medical procedures, recent outbreaks of *M. chelonae* cutaneous infections have occurred following tattooing and cosmetic procedures in otherwise healthy adults.^1^ Here, we report that *M. chelonae* can also cause localised soft‐tissue infections following a percutaneous inoculation injury in immunocompetent children. Such an infection should be considered, and mycobacterial cultures requested when a penetrating injury, possibly contaminated by either soil, water or plant material, is followed 4–8 weeks later by a cutaneous or soft tissue infection from which routine cultures fail to identify bacterial pathogens.^1^ This is especially true if there is no improvement after the wound has been drained and explored, any foreign body removed, and a course of empirical antibiotics completed.

Historically, *M. chelonae* has been combined with the more common *Mycobacterium abscessus* complex.^2^ Although we identified several studies reporting *M. chelonae* cutaneous and soft‐tissue infections (Table 1), these do not definitively confirm *M. chelonae* as the causative agent, due to either incomplete diagnostic work‐up or not reporting which identification testing was performed. Furthermore, with one exception,^3^ each of these reports provided few clinical details, and thus there is limited knowledge of soft‐tissue *M. chelonae* infections in immunocompetent children.

**Table 1:** *Mycobacterium chelonae* cutaneous and soft tissue infections reported in immunocompetent paediatric patients

Case	Underlying disease	Lesion site/s	Inoculation route	Treatment and outcome
14‐year old female^3^	Nil	Lower leg	Self‐inflicted injection of stagnant water	Clarithromycin and tobramycin, which was replaced by clofazimine for 2‐months Outcome: Uncertain due to continued self‐inflicted inoculation of sites on her leg
12‐year old female^4^	Nil	Cheek	Scratch from bathroom cabinet after a fall	Rifampicin, clarithromycin and ciprofloxacin for 6‐months Outcome: Resolution of lesion
20‐month old female^5^, †	Nil	Face	Nail scratch	Course 1: Oxacillin for 2‐weeks then incision and drainage. Course 2: Cefaclor 2‐weeks. Course 3: Erythromycin 6‐weeks and Rifampicin 2‐weeks Outcome: Resolution of lesions
3‐year‐old^6^, ‡	Not reported	Feet	Not reported	Amoxicillin‐clavulanate, trimethoprim‐sulfamethoxazole Outcome: Resolution of lesions
4‐year‐old^6^, ‡	Not reported	Hands and feet	Not reported	Clarithromycin for 12‐weeks and incision and drainage Outcome: Resolution of lesions
6‐year‐old^6^, ‡	Not reported	Foot	Not reported	Clindamycin Outcome: Not reported
2 children, ages unspecified^7^, ‡	Not reported	Not reported	Not reported	Not reported

† Not demonstrated to be either *M. chelonae* or a member of the *Mycobacterium abscessus* complex as validation methods were unavailable at the time of publication.

‡ Not clearly determined to be either *M. chelonae* or *M. abscessus* complex as the isolate was not investigated further by the testing laboratory or this was not reported by authors.

Since 1992, it has been possible to differentiate *M. chelonae* and *M. abscessus* from one another by employing restriction enzyme analysis of heat shock protein 65, which identifies differing intergenic sequences between them allowing each to be assigned to their own species. More recently, whole or partial genome sequencing can differentiate between these species, or as in our case matrix‐assisted laser desorption/ionisation time‐of‐flight mass spectrometry will also identify NTM species in clinical samples.^1^

Once identified, antibiotic susceptibility testing guides treatment regimens. A study in 2019 reported 96% of *M. chelonae* strains were susceptible to clarithromycin,^8^ while most isolates are also susceptible to cefoxitin, fluoroquinolones and tobramycin.^9^ The strain reported here showed susceptibility to azithromycin/clarithromycin and the aminoglycosides, amikacin and tobramycin, with intermediate susceptibility to moxifloxacin and imipenem. The choice of antibiotics for medical management is complicated by intrinsic antibiotic resistance. Combination therapy for 4–6 months is required as monotherapy risks failure from organisms also acquiring resistance during treatment.^1^ An additional challenge is that many of the antibiotics are poorly tolerated and need ongoing monitoring for evidence of toxicity. Finally, primary source control using debulking surgical debridement and washout techniques to remove any foreign bodies and drain abscesses remains critical for management of these patients.

Written informed consent was provided by the family of the patient.
